# Associations between community water fluoridation cessation and the prevalence of dental caries and fluorosis in Alrass city, Saudi Arabia

**DOI:** 10.3389/froh.2025.1508466

**Published:** 2025-04-07

**Authors:** Murad Alrashdi

**Affiliations:** Department of Orthodontic and Pediatric Dentistry, College of Dentistry, Qassim University, Burayadh, Saudi Arabia

**Keywords:** community water fluoridation, dental caries, dental fluorosis, public health, epidemiology

## Abstract

**Background:**

The relationships between fluoride exposure, dental caries, and fluorosis are well-known, but the long-term effects of changes in community water fluoridation practices remain unclear, particularly in Alrass city, Saudi Arabia.

**Aim:**

This study investigated how community water fluoridation cessation affected the prevalence of dental caries and fluorosis in Alrass City, Saudi Arabia.

**Design:**

This retrospective cross-sectional study included 568 participants aged 6–50 years, who were recruited through stratified random sampling in schools and public places. Clinical examinations were conducted using the DMFT index for caries and Dean's Fluorosis Index for fluorosis. Data analysis was performed to compare the difference in caries and fluorosis between age groups and between current and historical data across age groups.

**Results:**

Results showed significantly higher DMFT scores in adults (5.62) compared to children (3.98) (*p* < 0.0001). Dental fluorosis prevalence was higher in adults (41%) than children (18%) (*p* < 0.0001). Compared to historical data, mean DMFT scores significantly increased in both groups, while fluorosis prevalence decreased (*p* < 0.001). Subgroup analysis revealed lower DMFT scores in ages 6–12 compared to 19–50. Multivariate regression confirmed age as a significant predictor of DMFT scores (*p* < 0.001). Overall, caries prevalence increased and fluorosis decreased post-cessation of well water usage.

**Conclusion:**

These findings underscore the dual-edged nature of fluoride exposure through community water supply systems.

## 1 Introduction

Dental fluorosis, characterised by changes in the enamel ranging from barely noticeable white spots to severe staining and pitting, is a biomarker of excessive fluoride exposure during enamel formation (1). The aesthetic implications of this condition, particularly in the permanent incisors, are a significant cause for public health concern (2). However, the protective effect of fluoride against dental caries is well known. Fluoride aids enamel remineralisation and inhibits demineralisation, playing a crucial role in preventing dental caries (3).

Community water fluoridation, recognised by the Centers for Disease Control and Prevention as one of the ten great public health achievements of the 20th century, has been a cornerstone of dental caries prevention (4). Nonetheless, the relationship between fluoride exposure and dental health is complex. While low-to-moderate fluoride levels in drinking water significantly reduce the prevalence of caries, excessive exposure, particularly during the critical period of dental development in early childhood, can lead to dental fluorosis (1).

The relationships between fluoride exposure, dental caries, and fluorosis have been extensively studied; however, substantial knowledge gaps remain, particularly regarding the long-term effects of changes in community water fluoridation practices. Studies have primarily focused on the effects of introducing fluoridation, and less so on its cessation, especially in communities transitioning from natural high-fluoride water sources to lower-fluoride alternatives (5). Addressing this knowledge gap is critical, as the cessation of fluoridation presents unique challenges and potential risks to public dental health, particularly in areas with naturally high fluoride levels in the water. Moreover, while the global prevalence of dental fluorosis has been documented, there is a need for more localised studies that consider specific environmental and demographic factors influencing dental health outcomes (6).

Historically, Alrass City in Saudi Arabia has relied on well water, which often has a naturally high fluoride content and has been linked to a higher prevalence of dental fluorosis (7). However, in 2012, the city shifted to bottled water consumption, presumably with lower fluoride levels. This distinctive transition from well water to desalinated and bottled water provides a unique, natural opportunity to study the effects of fluoride in a localised setting, such as the prevalence of dental caries and fluorosis due to altered fluoride exposure levels. Understanding these local dynamics is essential for developing targeted public health strategies and guidelines. Historically, well water in Alrass City had a fluoride concentration of 1.6 ppm, which is above the recommended level of 0.7 ppm by World Health Organization (WHO) standards and other public health recommendations (7, 8).

In the context of global oral health, the balance between the protective effects of fluoride against caries and its potential to cause fluorosis remains delicate. The WHO guidelines emphasise the need for ongoing surveillance and research in this area (9). Therefore, this study examined how the cessation of high-fluoride well water usage affected the prevalence of dental caries and fluorosis in Alrass City, aiming to provide a more nuanced understanding of the dual role of fluoride in dental health and the optimal fluoride levels in community water supplies. In addition, by focusing on a location with a distinct water fluoridation history, we aimed to inform public health policies in Alrass City and other cities globally that are in similar situations where shifts in water fluoridation practices have occurred or are being considered. Overall, this study sought to contribute to the ongoing debate regarding the optimal fluoride levels in community water supplies and offer insights that could inform policy and practice, both locally and internationally.

## 2 Materials and methods

### 2.1 Study design and duration

This cross-sectional population-based study was designed to evaluate the effect of the cessation of community water fluoridation on the prevalence of dental caries and fluorosis in Alrass City, Saudi Arabia. This design was selected because of its effectiveness in assessing the prevalence of conditions in a specific population at a given time (10). This study was conducted from June 2022 to June 2023. This period was chosen to ensure adequate time for participant recruitment, data collection, and analysis.

### 2.2 Sample size

The sample size for this study was calculated using the following parameters: (1) Expected prevalence: Pre-exposure prevalence; 70%–90% for primary dentition and 69%–84% for permanent dentition was determined from previous studies. Post-exposure prevalence was unknown, but expected to increase. (2) Given the high pre-exposure prevalence, we aimed to detect a minimum increase of 10% in prevalence. We set the desired power at 90% (*β* = 0.10) to ensure a high probability of detecting a true difference if one exists. We used a two-sided significance level of 5% (*α* = 0.05). Using these parameters, we calculated the sample size using the following formula for comparing two proportions: $n\;=\;{\left(\begin{array}{c} \frac{\left(Z_{\frac{\alpha }{2}}+\;Z_{\beta }\right)}{\;}\\ \left(\;p_{1}\text{–}\;p_{2}\right) \end{array}\right)}^{2}[\;p_{1}(1\;\text{–}\;p_{1})+\;p_{2}(1\;\text{–}\;p_{2})]$ Where: Z*α*/2 = 1.96 (for *α* = 0.05), Z*β* = 1.28 (for 90% power), *p*_1_ = 0.90 (using the highest pre-exposure prevalence to be conservative), *p*_2_ = 1.00 (assuming a 10% increase, capped at 100%). To account for the stratified sampling design, we applied a design effect of 1.5. To account for potential non-response and to ensure adequate representation across age groups and dentition (primary and permanent), we increased the sample by 100%, bringing our target sample size to 284 per group (children and adults). Thus, our total target sample size was 568. By using the highest pre-exposure prevalence, we ensured that our sample size is large enough to detect changes even in the highest prevalence scenario. A 10% increase in prevalence was considered statistically significant, especially given the already high baseline prevalence. The 100% increase in sample size after applying the design effect allowed for: (a) Subgroup analyses (e.g., comparing primary vs. permanent dentition) (b) Potential non-response or incomplete data (c) Maintaining power even if the actual prevalence increase is smaller than 10%.

### 2.3 Study population and sampling technique

The study included two demographic groups: children aged 6–17 years and adults aged 18–50 years. Each of these demographics was selected based on their unique exposure history to the community water sources in Alrass City, specifically well water and its subsequent cessation. This differentiation is crucial for understanding the differential effects of fluoride exposure on dental health across various life stages.

#### 2.3.1 Group 1 (children aged 6–17 years)

This group represents the younger population that has primarily been exposed to bottled water following the cessation of well water usage in Alrass City in 2012. Children in this age range are of particular interest because they were either not born or very young at the time of the water source transition. This demographic provides critical insights into the dental health effects in a population with limited to no direct exposure to the higher fluoride levels typically found in well water. Their dental health status, particularly the prevalence of dental caries and the absence or minimal presence of fluorosis, indicates the long-term effects of reduced fluoride exposure from an early age.

#### 2.3.2 Group 2 (adults aged 18–50 years)

This group comprises individuals who have consumed well water for a substantial portion of their lives before the shift to bottled water. As such, they will likely have been exposed to higher fluoride levels during their critical developmental years. The prevalence of dental fluorosis among this group is an essential focus, as it reflects the historical exposure to high fluoride levels in the well water. Additionally, observing the prevalence of dental caries in this group provides a comparative perspective against the younger demographic, offering a comprehensive view of how changes in water fluoridation over time affect different age groups in terms of both dental caries and fluorosis. By analysing these two groups, the study aims to comprehensively understand the full spectrum of effects of fluoride exposure, from a younger generation with minimal well water exposure to an older generation with considerable exposure. This approach allows for a nuanced analysis of the changes in dental health trends related to the termination of well water usage in Alrass City.

#### 2.3.3 Inclusion and exclusion criteria

Residents aged 6–50 years born in Alrass City who never migrated to another city were included. Residents of Alrass City not born in the city, as well as individuals with medical conditions that could affect their oral health, were excluded. In addition to excluding residents not born in Alrass City and individuals with medical conditions affecting oral health, participants with conditions that could alter fluoride excretion, such as kidney disease, were also excluded from this study. This was done to minimize confounding variables that could potentially influence the study's outcomes on dental caries and fluorosis.

#### 2.3.4 Sampling method

This study employed a stratified random sampling technique. Schools were defined as the primary sampling sites for children, whereas public places, such as shopping malls, hospitals, and academic facilities, served as sampling sites for adults. The population was stratified based on two key factors: (a) Age groups: Children (6–17 years) and Adults (18–50 years) (b) Geographical regions: North, South, East, and West of Alrass City. The schools were stratified by region (north, west, east, and south of Alrass City), and a random sample of schools was selected from each region to ensure adequate regional representation. Similarly, public places were chosen based on their demographic distribution and accessibility.

#### 2.3.5 Participant recruitment process

Schools were randomly selected from each geographical stratum (north, south, east, and west of Alrass City) using a random number generator. The sampling frame was established using a comprehensive list of all primary and secondary schools in Alrass City, which was obtained from the local education authorities. This ensured that the recruitment process covered the full range of schools catering to children aged 6–17 years. The four geographical zones were defined based on the administrative divisions of the city, including wards, to ensure a representative distribution of schools across the entire city.

To account for differences in the number of schools within each geographical zone, proportional stratified sampling was employed. The probability of selecting a school was weighted based on the total number of schools in each zone, ensuring fair representation of both densely and sparsely populated areas. Within each selected school, classes across different grade levels were randomly chosen. Individual students were then selected using a random number generator applied to the class lists, ensuring each student had an equal probability of selection.

This multi-stage sampling approach was designed to capture the diversity of the population while minimizing selection bias. By integrating administrative boundaries and stratifying by geographical zones, this method ensured that the final sample was representative of children in Alrass City across all regions and age groups.

Adult participants were recruited directly from selected public places such as shopping malls, hospitals, and academic facilities, as these locations ensured access to a diverse and representative sample of the city's population. This approach was chosen to facilitate participant accessibility and compliance, considering potential social and cultural factors. For instance, accessing participants in private homes, particularly women in purdah, posed challenges due to cultural norms and privacy considerations. To ensure inclusivity, recruitment sites were selected to encompass locations frequently visited by both men and women, and recruitment sessions were conducted during different times of the day to capture a wide demographic.

While suburban and slum areas were not specifically targeted, the inclusion of public places ensured that individuals from diverse socioeconomic backgrounds were reached. This strategy was designed to balance logistical feasibility with the goal of obtaining a representative sample of adults in Alrass City.

To ensure a diverse and representative sample, multiple recruitment sessions were conducted at different times and days across all four geographical divisions (north, south, east, and west) of Alrass City. A predetermined number of recruitment sites were selected in each division, proportionate to the population density and the number of educational institutions, malls, and hospitals within each zone. For example, public places such as shopping malls and hospitals were chosen based on their accessibility and frequency of use by diverse demographics, while schools were stratified to represent both primary and secondary institutions across all regions.

The number of recruitment sites in each division was determined through proportional stratified sampling, ensuring equal representation of the city's geographic and demographic diversity. This method ensured that recruitment efforts captured participants from all socioeconomic and cultural backgrounds within the city.

### 2.4 Data collection and measurement

#### 2.4.1 Exposure assessment

To assess fluoride exposure, we relied on existing data and participant-reported information, acknowledging the limitations of direct measurement in this cross-sectional study. Historical fluoride levels were obtained from previous studies and government reports, which indicated a range of 0.23–1.6 mg/L in groundwater sources for the Al-Qassim region, including Alrass (7). Current exposure levels were estimated based on reported ranges of 0.01–0.5 mg/L in the distribution network. Specifically, the reported fluoride levels reflect the concentrations found in desalinated and bottled water commonly distributed in the region following the cessation of well water use.

We administered a detailed questionnaire to participants to gather information on their primary drinking water sources. The rationale for this assessment method was to provide a reasonable estimate of fluoride exposure within the constraints of a cross-sectional study design. Historical DMFT scores and prevalence of fluorosis were also retrieved from previous studies (9).

#### 2.4.2 Clinical examinations

The DMFT index was chosen as our primary measure for dental caries due to its widespread use and acceptance in epidemiological studies. This index provides a cumulative measure of caries experience. DMFT is recommended by the World Health Organization (WHO) for oral health surveys, allowing for comparisons with other studies. Each tooth was scored as decayed (D), missing due to caries (M), or filled (F), following WHO criteria. The dental fluorosis index used was the Dean's Fluorosis Index, which categorises fluorosis into levels ranging from ‘questionable’ to ‘severe’ based on the visual and tactile examination (9). The clinical examination was carried out by two dentists using sterilized packaged hand instruments under natural lighting conditions. The subjects were seated upright on a portable chair during the dental examination. Dental examinations were conducted in various locations based on the recruitment site. For participants recruited from schools and hospitals, examinations were performed in designated rooms or areas within the facilities. In shopping malls, a portable dental station was set up in a quiet, semi-private area to ensure participant comfort and privacy. Participants were seated upright on a portable dental chair, and examinations were performed under natural lighting conditions using sterilized hand instruments. This setup was designed to maintain consistency and reliability across all recruitment locations while ensuring adherence to ethical and clinical standards.

Examiners underwent rigorous training and calibration. Inter-examiner reliability was assessed using intraclass correlation coefficients (ICC), with a minimum acceptable ICC of 0.85. Visual-tactile examinations were conducted using a dental mirror and WHO CPI probe.

### 2.5 Data analysis

The dataset was divided into two age groups: 6–17 years and 18–50 years. To analyse the difference in DMFT scores between these groups, an independent-samples t-test was performed. A chi-square test of independence was conducted to examine the association between age group and the presence of dental fluorosis. The chi-square test assessed whether the proportion of individuals with fluorosis differed significantly between the two age groups. Subgroup analysis was performed to explore potential differences in the relationship between DMFT scores, and fluorosis across various subgroups of smaller age ranges. To account for sex as a confounder, we also performed multivariate regression. A comparative analysis was also performed between our DMFT scores and fluorosis and historical values reported in literature. *P* value < 0.05 was considered statistically significant. All statistics were performed using R program.

### 2.6 Ethical considerations and compliance with reporting guidelines

This study was conducted in accordance with the principles outlined in the Declaration of Helsinki. Our institution's review board provided ethical approval for the research, ensuring that all procedures involving human participants were in line with the ethical standards of the institutional and national research committees. Informed consent was obtained from all individual participants involved in the study. For participants under the age of 18 years, informed consent was duly obtained from their legal guardians. The confidentiality of participant data was rigorously maintained throughout the research process, and all data were anonymised to protect participant privacy. Furthermore, the design, implementation, and reporting of this cross-sectional study adhered to the Strengthening the Reporting of Observational Studies in Epidemiology (STROBE) guidelines.

In addition to obtaining ethical approval from the Institutional Review Board and informed consent from participants and their legal guardians, we also obtained formal permission from the school authorities prior to conducting recruitment and examinations. Letters outlining the study objectives and procedures were sent to the administrative offices of each selected school, and written approval was received before initiating participant recruitment and data collection.

## 3 Results

### 3.1 Demographic characteristics

The study included a total of 568 participants, divided into two age groups: 251 children aged 6–17 years (44% of the total sample) and 317 adults aged 18–50 years (56% of the total sample). The distribution of participants by sex was fairly balanced, with 127 males and 124 females in the children's group, and 147 males and 170 females in the adult group, resulting in an overall distribution of 274 males and 294 females. The mean age of participants in the children's group was 11 years (±3.6 years), while the mean age in the adult group was 34 years (±10 years), with an overall average age of 23 years for the entire study population. Notably, all participants (100%) in both age groups were born and raised in Alrass City, ensuring a consistent demographic background across the study sample (Table 1).

**Table 1:** Demographic characteristics of the study participants.

Demographic feature	Children (6–17 years)	Adults (18–50 years)	Total
Number of participants	251 (44%)	317 (56%)	568
Sex (Male/Female)	127/124	147/170	274/294
Mean age (years)	11 ± 3.6	34 ± 10	23
Born and raised in Alrass city (%)	100	100	100

### 3.2 Prevalence of dental caries

The older age group (18–50 years) demonstrated a significantly higher mean DMFT score of 5.62 compared to 3.98 in the younger group (6–17 years). This difference is statistically significant (*p* < 0.0001), indicating a higher burden of dental caries and related issues in the older population (Table 2). The mean DMFT scores significantly increased in both groups after the cessation of well water usage compared to the historical data (both *p* < 0.001), indicating a substantial increase in the prevalence of dental caries (Table 3).

**Table 2:** Comparison of mean DMFT scores and proportion of fluorosis between age 6–17 and age 18–50.

	Age 6–17 years	Age 18–50 years	95% CI	*P*-value
DMFT (Mean ± SD)	3.98 ± 1.09	5.62 ± 1.15	1.82–1.45	<0.0001
Fluorosis (%)	45 (18%)	130 (41%)	–	<0.0001

**Table 3:** Prevalence of dental caries.

Age group	Mean DMFT score after cessation	Historical mean DMFT scores	95% CI	*P*-value
6–12 years	3.5	2.1	0.83–1.97	<0.0001
13–18 years	4.2	2.7	0.77–2.23	<0.001
19–50 years	5.6	3.9	0.82–2.58	<0.001

DMFT, decayed, missing, and filled teeth.

### 3.3 Prevalence of dental fluorosis

The prevalence of dental fluorosis was markedly higher in the older group, affecting 41% of individuals compared to 18% in the younger group, which was also statistically significant (*p* < 0.0001). The prevalence of dental fluorosis significantly decreased among children (*p* < 0.001) and moderately but significantly decreased among adults (*p* = 0.005) compared to the historical data (Table 4).

**Table 4:** Prevalence of dental fluorosis.

Age group	Prevalence of dental fluorosis after cessation (%)	Historical prevalence of dental fluorosis (%)	*P*-value
6–17 years	45 (18%)	45	<0.001
18–50 years	130 (41%)	55	0.005

### 3.4 Subgroup analyses

The youngest group (6–12 years) had significantly lower DMFT scores compared to the oldest group (19–50 years), indicating fewer dental caries. Interestingly, the fluorosis percentage in the 6–12 years age group (26%) was slightly higher than in the 19–50 years age group (23%), which was statistically significant. There was no significant difference in DMFT scores between the 6–12 years and 13–18 years age groups, suggesting similar caries experiences in childhood and adolescence. The fluorosis percentage in the 13–18 years age group (35%) was dramatically higher than in the 6–12 years age group (26%). These findings suggest that young children (6–12 years) who had less exposure to high-fluoride well water do not have significantly higher caries rates compared to adolescents (13–18 years). However, they do have significantly lower DMFT scores compared to adults (19–50 years), which could be attributed to less cumulative exposure to caries risk factors over time (Table 5).

**Table 5:** Subgroup analysis of mean DMFT scores and fluorosis for subgroup of age 6–12.

	Age 6–12 years	Age 19–50 years	95% CI	*P*-value
DMFT (Mean ± SD)	3.93 ± 1.12	5.70 ± 1.07	1.99–1.55	<0.0001
Fluorosis (%)	65 (26)	73 (23)	–	<0.0001
	Age 6–12	Age 13–18	95% CI	*P*-value
DMFT (Mean ± SD)	3.93 ± 1.12	4.02 ± 1.09	−0.36–1.73	0.489
Fluorosis (%)	65 (26)	85 (35)	–	<0.0001

### 3.5 Multivariate regression analysis

The multivariate regression analysis revealed that age group is a significant predictor of DMFT scores (*p* < 0.001), with a large effect size, accounting for approximately 38.7% of the variance in DMFT scores. The influence of sex on DMFT scores is minimal and not statistically significant, suggesting that the difference in DMFT scores between age groups is not confounded by sex. The model as a whole explained 39.0% of the variance in DMFT scores, indicating a good fit.

## 4 Discussion

This study evaluated the associations between the cessation of water fluoridation and the prevalence of dental caries and fluorosis, particularly in children and adolescents who are most vulnerable to these changes. We found that the prevalence of dental caries significantly increased, while the prevalence of dental fluorosis decreased, after the cessation of well water usage. The risks for both these diseases followed a similar trend. These findings highlight significant shifts in the incidence of dental caries and fluorosis in Alrass City following the cessation of community water fluoridation. The increased incidence of dental caries, indicated by elevated DMFT scores after cessation, aligns with similar observations in other populations that have experienced changes in fluoride exposure. Our findings extend this narrative by demonstrating a definite association between reduced fluoride exposure and increased risk of dental caries, emphasising the protective role of fluoride in dental health.

A nuanced relationship between fluoride exposure and oral health outcomes has been observed in various settings worldwide, underlining the need for localised studies, such as the present study in Alrass City. For instance, a long-term study compared the incidence of caries and fluorosis in children in two cities in New York, USA, Newburgh and Kingston, who received fluoridated and non-fluoridated water, respectively. Although children in Newburgh had significantly fewer caries, the incidence of mild dental fluorosis was higher (10), highlighting the delicate balance between the benefits and risks of fluoride exposure. In contrast, an increase in the incidence of dental caries was observed in Vancouver following the termination of water fluoridation (11). This cessation led to an increase in the incidence of caries, particularly among children, underscoring the protective role of fluoridated water against dental decay. These findings are consistent with those of a previous study indicating that fluoride significantly reduces the incidence of dental caries when optimally dosed in community water supplies (12).

The results of the present study align with the global pattern of changes in fluoride exposure through community water supplies tangibly impacting public oral health. Examining the effects of shifting from well water (naturally high in fluoride) to desalinated and bottled water (typically low in fluoride) in Alrass City expands the understanding of these dynamics in different cultural and geographical contexts. The fluctuating patterns of dental caries and fluorosis in response to changes in community water fluoridation observed in various cities worldwide accentuate the complexity of the influence of fluoride on public oral health. These case studies illustrate that the effects of altering fluoride levels in community water are significant, variable, and require careful, context-specific investigation. The decision to shift from well to desalinated and bottled water in Alrass City and its subsequent effects on dental health resonate with broader public health debates. The WHO guidelines on drinking water quality acknowledge the complexity of managing fluoride levels to prevent dental caries while avoiding excessive exposure that leads to fluorosis (13). The experience in Alrass City is a valuable case study on this global discourse, offering insights into the real-world implications of such public health decisions. Considering the global discussion and diverse experiences of communities adjusting their water fluoridation practices, localised research is warranted to understand the specific effects in different geographical and cultural settings.

Herein, a marked decrease in the prevalence of dental fluorosis, especially among younger populations, was a notable outcome of reduced fluoride exposure. This reduction is consistent with experiences in cities such as Newburgh, where a decrease in fluoride concentration led to a lower incidence of fluorosis (10). Our study adds to this body of evidence; we suggest that while a reduction in fluoride levels in drinking water can mitigate the risk of fluorosis, it simultaneously increases the risk of dental caries. Furthermore, our results stress the importance of considering local environmental and demographic factors while framing policies related to fluoride. The demographic differences in the prevalence of fluorosis and caries within our study population suggest that individual- and community-level factors, such as age, dietary habits, and access to dental care, may considerably influence the outcomes of changes in water fluoridation. This observation is supported by research that emphasises the need for customised approaches to fluoride management (12). These findings suggest a compelling need for the ongoing monitoring and adjustment of fluoride levels in community water supplies, coupled with targeted public health initiatives, including educational programs on oral hygiene, use of fluoride toothpaste, and professional dental care, especially in communities experiencing changes in water-fluoridation practices.

This study had some limitations. Although a cross-sectional design is effective for determining prevalence, it does not establish causality. Longitudinal studies are required to elucidate the direct effects of changes in fluoride exposure over time. Additionally, factors such as nutrition, socioeconomic status, and individual oral hygiene practices, which can influence dental health outcomes, were not extensively controlled in this study, and warrant further investigation.

In conclusion, this study provides critical insights into the consequences of altering community water-fluoridation practices. The observed increase in the incidence of dental caries and decrease in the incidence of fluorosis in Alrass City after the cessation of well water usage contributes to the global discourse on optimal fluoride management in public health. These findings highlight the need for a balanced approach to fluoride exposure that is tailored to the specific needs and characteristics of individual communities.

## 5 Why this paper is important to paediatric dentists

• Associations between the cessation of water fluoridation and the prevalence of dental caries and fluorosis were identified, particularly in children and adolescents.
• Although reduced fluoride exposure appeared to reduce fluorosis, the concomitant increase in dental caries highlights the need for balanced fluoride exposure to maintain optimal dental health.
• These results are pivotal for public health policy and emphasise the need to carefully consider community water fluoridation.

## Data Availability

The raw data supporting the conclusions of this article will be made available by the authors, without undue reservation.
